# Renal sarcoidosis associated with certolizumab pegol treatment for psoriatic arthritis

**DOI:** 10.1093/omcr/omac133

**Published:** 2022-12-16

**Authors:** Ryan M Hum, Durga A Kanigicherla, Pauline Ho

**Affiliations:** The Kellgren Centre for Rheumatology, Manchester University NHS Foundation Trust, Manchester, UK; NIHR Manchester Biomedical Research Centre, The University of Manchester, Manchester, UK; Manchester Institute of Nephrology and Transplantation, Manchester University NHS Foundation Trust, Manchester, UK; The Kellgren Centre for Rheumatology, Manchester University NHS Foundation Trust, Manchester, UK; NIHR Manchester Biomedical Research Centre, The University of Manchester, Manchester, UK

## Abstract

We present a case of certolizumab-associated renal sarcoidosis, the first reported case in a patient with psoriatic arthritis (PsA) that was effectively treated with corticosteroids. A 55-year-old Caucasian man with PsA diagnosed at age 47 and plaque psoriasis since his early twenties was on certolizumab pegol (CZP) for 7 months before presenting to the emergency department with seizures and renal failure. A renal biopsy confirmed renal sarcoidosis. His CZP therapy was stopped, and after several months taking prednisolone at a reducing regime, his renal function improved, and his PsA remained under control. When considering further treatment options for his PsA keeping in mind that other drugs, especially tumour necrosis factor-alpha inhibitors, have been reported to be associated with sarcoidosis, tofacitinib was considered to be a future treatment option acceptable to the patient, given current National Institute for Health and Care Excellence guidelines approving its use in PsA and the lack of reports of tofacitinib-associated sarcoidosis in the literature.

## INTRODUCTION

Sarcoidosis is a systemic granulomatous disease, where environmental triggers are thought to provoke disease in genetically predisposed individuals [1]. Drug-induced sarcoidosis-like reactions (DISR) are systemic granulomatous reactions that are indistinguishable from sarcoidosis, which occur in temporal relationship with the initiation of an offending drug [2]. Despite numerous reports, it is not clear if these drugs cause exacerbating subclinical cases or cause distinct conditions from sarcoidosis [2].

Treatment of psoriatic arthritis (PsA) often involves immunomodulatory therapy such as biological disease-modifying antirheumatic drugs (DMARDs) including tumour necrosis factor-alpha inhibitors (TNFi), which have been reported to cause DISR [2, 3]. Paradoxically, TNFi have been used in addition to corticosteroids and DMARDs to treat severe cases of sarcoidosis [4]. Certolizumab pegol (CZP) is an Fc-free PEGylated TNFi approved for the treatment of PsA by the National Institute for Health and Care Excellence (NICE) [5].

We present the first reported case of renal sarcoidosis occurring following CZP treatment in a patient with PsA.

## CASE REPORT

A 55-year-old Caucasian man, born in the UK, presented to the emergency department with a seizure and was found to have a stage-3 acute kidney injury. He was diagnosed with PsA at age 47 and plaque psoriasis at 20 with no relevant medical, family or travel history. He did not take non-steroidal anti-inflammatory or other prescription medications. For PsA, he was initially treated with methotrexate (MTX), but this was stopped due to nausea. After failing on sulfasalazine, he was started on adalimumab with a second trial of MTX. Due to persistent disease activity, he opted to try etanercept to achieve better disease control. After 1 year to achieve better disease control he opted to switch to CZP.

He was on the CZP treatment for 7 months before presenting with seizures and renal failure. Whilst on CZP he did not have any disease flares, suggesting an at least partial response. He had severe renal dysfunction (estimated glomerular filtration rate [eGFR] of 8 ml/min/1.73 m^2^), but low-level proteinuria (uPCR of 47 mg/mmol). His seizure was attributed to uraemia and hypertension, which were the result of renal failure. Emergency dialysis was considered, but deemed not required after resuscitation. A renal biopsy was performed, which showed features of non-necrotizing interstitial nephritis, with well-formed granulomas and multiple giant cells, consistent with renal sarcoidosis. There were no episodes of hypercalcaemia.

Further investigations included a computed tomography scan of the thorax, abdomen and pelvis, which showed no lymphadenopathy, a renal ultrasound, which was unremarkable, elevated serum angiotensin-converting enzyme levels of 77 IU/l (reference range 20–70) and a magnetic resonance scan of the brain showed small vessel ischaemic changes. Screening for infection was performed including tuberculosis (gamma interferon), urinary legionella/pneumococcal antigen, hepatitis B/C/HIV, vitamin D and urine culture and sediment, all of which were unremarkable. No extra-renal organ involvement was found. Screening for autoimmune conditions with autoantibody testing was negative.

**Figure 1:**
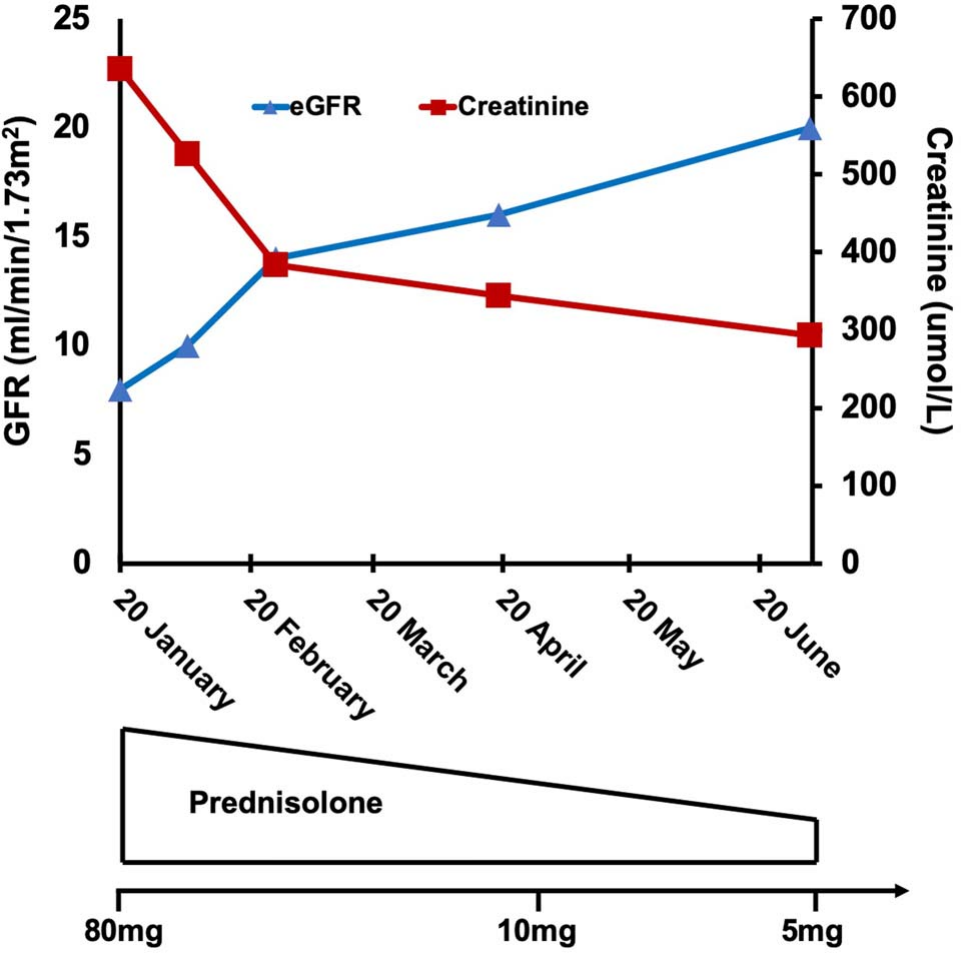
Clinical course over 6 months highlighting treatment regime with a tapering dose of prednisolone and gradual improvement in renal function.

He was started on prednisolone 80 mg daily, and a modest improvement in creatinine was observed from 636 μmol/l (eGFR 8 ml/min/1.73 m^2^) on admission to 526 μmol/l (eGFR 10 ml/min/1.73 m^2^) on discharge a week later. He did not require renal replacement therapy during admission. The dose of prednisolone was tapered over time to 5 mg per day with a stable creatinine of 262 μmol/l 21 months after his initial presentation, (eGFR 22 ml/min/1.73 m^2^) (Fig. 1 and Table 1). His PsA remained well controlled with corticosteroids alone.

**Table 1:** Measurements of the patient’s renal function prior to and after emergency presentation

Time from described emergency presentation (months)	Creatinine (μmol/l)	eGFR (ml/min/1.73m^2^)
−40	81	>60
−35	89	>60
−33	103	68
−28	108	65
−26	98	73
−17	138	48
−16	142	47
−15	146	45
0	636	8
1	526	9
2	403	14
3	331	17
4	336	17
6	293	20
9	296	20
10	256	23
11	264	22
14	294	20
21	262	22

## DISCUSSION

CZP-associated sarcoidosis is a rare adverse event but is increasingly reported [5, 6]. A literature review found 87 published case reports of TNFi-associated sarcoidosis, 85 of which were in patients receiving etanercept, adalimumab and infliximab and two associated with CZP [3, 5, 6].

In the first case, a 45-year-old Caucasian woman with HLA-B27-positive nonradiographic axial spondyloarthritis developed renal insufficiency and mediastinal lymphadenopathy following 6 months of CZP therapy. Renal and bronchial biopsies were consistent with sarcoidosis. The patient was successfully treated with prednisolone, MTX and secukinumab [5].

In the second case, a 69-year-old woman with rheumatoid arthritis developed fatigue, appetite loss and low-grade fever after 6 years of CZP therapy. She was found to have granulomatous uveitis, mediastinal lymphadenopathy, a subcutaneous nodule on her right knee and micronodules in the lungs. A video-assisted thoracoscopic biopsy showed noncaseous epithelioid cell granulomas consistent with sarcoidosis. She was successfully treated with prednisolone and abatacept without recurrence of sarcoidosis [6].

In all three reported cases of CZP-associated sarcoidosis, discontinuation of CZP and corticosteroid treatment resulted in resolution.

When considering future treatment options for PsA, there remains concerns about risk of DISR with other targeted therapies. Secukinumab an IL-17 target that is NICE approved for PsA has been reported in one published case to have caused secukinumab-associated sarcoidosis [4, 7]. Tofacitinib is a Janus kinase (JAK) inhibitor that has recently been approved by NICE for the treatment of PsA, and to date there have been no reports of JAK inhibitor-associated sarcoidosis. Therefore, in this case tofacitinib was considered a suitable future treatment option for the management of the patients PsA [8].

## References

[1] Valeyre D, Prasse A, Nunes H, Uzunhan Y, Brillet PY, Müller-Quernheim J. Sarcoidosis. Lancet 2014;383:1155–67. 10.1016/S0140-6736(13)60680-7. 24090799

[2] Chopra A, Nautiyal A, Kalkanis A, Judson MA. Drug-induced sarcoidosis-like reactions. Chest 2018;154:664–77. 10.1016/j.chest.2018.03.056. 29698718

[3] Daïen CI, Monnier A, Claudepierre P, Constantin A, Eschard JP, Houvenagel E et al. Sarcoid-like granulomatosis in patients treated with tumor necrosis factor blockers: 10 cases. Rheumatology 2009;48:883–6. 10.1093/rheumatology/kep046. 19423648

[4] Koda K, Toyoshima M, Nozue T, Suda T. Systemic sarcoidosis associated with certolizumab pegol treatment for rheumatoid arthritis: a case report and review of the literature. Intern Med 2020;59:2015–21. 10.2169/internalmedicine.4275-19. 32389943 PMC7492107

[5] National Institute for Health and Care Excellence . (2017). Certolizumab pegol and secukinumab for treating active psoriatic arthritis after inadequate response to DMARDs [NICE Technology appraisal guidance TA445]. https://www.nice.org.uk/guidance/ta445.

[6] Toussirot E, Bernard C, Bossert M. Safety of the use of anti-IL17A treatment in a patient with certolizumab-induced sarcoidosis. Clin Exp Rheumatol 2019;37:344–5. 30873944

[7] Nyckowski T, Ceilley R, Wilson J. Sarcoidosis developing during secukinumab therapy: case report. J of Skin 2017;1:95–9. 10.25251/skin.1.2.7.

[8] National Institute for Health and Care Excellence . (2018). Tofacitinib for treating active psoriatic arthritis after inadequate response to DMARDs. [NICE Technology appraisal guidance TA543]. https://www.nice.org.uk/guidance/ta543. 41533816

